# Galectin‐3 promotes CXCR2 to augment the stem‐like property of renal cell carcinoma

**DOI:** 10.1111/jcmm.13860

**Published:** 2018-09-24

**Authors:** Chang‐Shuo Huang, Shye‐Jye Tang, Mei‐Hsuan Lee, Chien‐Chih Chang Wang, Guang‐Huan Sun, Kuang‐Hui Sun

**Affiliations:** ^1^ Department of Biotechnology and Laboratory Science in Medicine National Yang‐Ming University Taipei Taiwan; ^2^ Institute of Marine Biotechnology National Taiwan Ocean University Keelung Taiwan; ^3^ Division of Urology Department of Surgery Tri‐Service General Hospital and National Defense Medical Center Taipei Taiwan; ^4^ Department of Education and Research Taipei City Hospital Taipei Taiwan

**Keywords:** cancer stem cells, CXCR2, galectin‐3, renal cell carcinoma

## Abstract

Although targeted therapy is usually the first‐line treatment for advanced renal cell carcinoma (RCC), some patients can experience drug resistance. Cancer stem cells are tumour‐initiating cells that play a vital role in drug resistance, metastasis and cancer relapse, while galectins (Gal) participate in tumour progression and drug resistance. However, the exact role of galectins in RCC stemness is yet unknown. In this study, we grew a subpopulation of RCC cells as tumour spheres with higher levels of stemness‐related genes, such as Oct4, Sox2 and Nanog. Among the Gal family, Gal‐3 in particular was highly expressed in RCC tumour spheres. To further investigate Gal‐3's role in the stemness of RCC, lentivirus‐mediated knockdown and overexpression of Gal‐3 in RCC cells were used to examine both in vitro and in vivo tumorigenicity. We further assessed Gal‐3 expression in RCC tissue microarray using immunohistochemistry. Upon suppressing Gal‐3 in parental RCC cells, invasion, colony formation, sphere‐forming ability, drug resistance and stemness‐related gene expression were all significantly decreased. Furthermore, CXCL6, CXCL7 and CXCR2 were down‐regulated in Gal‐3‐knockdown tumour spheres, while CXCR2 overexpression in Gal‐3‐knockdown RCC restored the ability of sphere formation. Gal‐3 overexpression in RCC promoted both in vitro and in vivo tumorigenicity, and its expression was correlated with CXCR2 expression and tumour progression in clinical tissues. RCC patients with higher co‐expressions of Gal‐3 and CXCR2 demonstrated a worse survival rate. These results indicate that highly expressed Gal‐3 may up‐regulate CXCR2 to augment RCC stemness. Gal‐3 may be a prognostic and innovative target of combined therapy for treating RCC.

## 1 INTRODUCTION

Renal cell carcinoma (RCC) includes a heterogeneous group of primary kidney adenocarcinomas.^1^ Approximately one‐third of all patients have a locally advanced disease upon diagnosis. Furthermore, nearly 30% of patients who undergo nephrectomy for localized RCC eventually develop tumour recurrence.^2^ The five‐year survival rate of metastatic RCC is only 10% due to patients’ resistance to the currently available therapies. Although immunotherapy is one of the first‐line therapy for treating metastatic clear cell RCC (ccRCC), its overall efficacy rate is restricted by its toxicity. Molecular targeting drugs, including tyrosine kinase and mTOR inhibitors, have been approved for treating advanced RCC.^1^, ^2^ Nevertheless, long‐lasting treatment responses cannot be achieved, and the overall survival rate is still poor due to drug resistance.

Cancer stem cells (CSCs) may contribute to drug resistance in human solid tumours.^3^, ^4^ The frequency of functionally defined CSCs varies among different patients. With self‐renewal as one of their hallmarks, CSCs can initiate tumour formation and metastasis. Furthermore, CSCs can be identified by in vitro sphere‐forming assays and common cell surface markers, especially CD133 and CD44. CSCs can express ATP‐binding cassette (ABC) transporters to become more resistant to chemotherapy compared to the bulk of a tumour cell mass.^3^, ^4^ The tumour microenvironment supports cancer progression and CSC formation through growth factors, cytokines and chemokines. For example, within the tumour microenvironment, endothelial cells produce angiocrine factors and myofibroblasts secrete the stem cell factor, CXCL12 and Wnt to modulate the stemness of CSCs.^5^ Another critical component of the tumour microenvironment, galectin can control immune surveillance and aid tumour metastasis.^6^

Galectins (Gals) are galactoside‐binding lectins that contain conserved carbohydrate‐recognition domains (CRDs) to bind β‐galactose. According to their structural features, galectins are classified into the following three categories: prototype, chimera type and tandem‐repeat type. The chimera galectin type has only one member, Gal‐3,^7^ whose expression is required for initiating the transformed phenotype of tumours by interacting with oncogenic Ras.^8^ The Gal‐3‐RAS interaction promotes the RAS anchorage to the plasma membrane, which results in the constitutive activation of phosphatidylinositol 3‐kinase and Raf‐1.^9^ The tumorigenic potential of Gal‐3 may also function through binding with β‐catenin or transcriptional factors to increase the expressions of cyclin D and c‐MYC and augment cell cycle progression.^10^ Furthermore, intracellular Gal‐3 inhibits cell death induced by cisplatin and paclitaxel, thus contributing to cancer cells’ drug resistance and CSC formation.^11^, ^12^ Extracellular Gal‐3 has in vitro angiogenic activity by inducing the migration of endothelial cells.^13^

Increased protein levels of Gal‐3 are correlated with the poor survival of various cancers, including leukaemia, lymphomas, breast cancer and thyroid cancer.^6^ Gal‐3 was overexpressed in RCC patients with distant metastasis.^14^ While chemokines and their receptors influence the initiation and progression of tumours in the tumour microenvironment, their role in the Gal‐3‐promoted CSC formation and drug resistance of RCC remains unclear.^5^ In this study, we found that Gal‐3 was highly expressed in the CSCs of RCC, as well as the clinical tissues of advanced RCC. Silencing Gal‐3 in RCC cells decreased CSC formation, drug resistance and CXCR2, while CXCR2 overexpression in Gal‐3‐knockdown cells restored the tumorigenesis ability. Our results indicate that highly expressed Gal‐3 may enhance the stemness property of RCC by promoting CXCR2.

## 2 MATERIALS AND METHODS

### 2.1 Cell lines

We obtained the human RCC cell lines Caki‐1 and ACHN (VHL wild type) from the American Type Culture Collection. We purchased the human RCC cell line A‐498 (VHL mutation) from Bioresource Collection and Research Center (BCRC; Hsinchu, Taiwan). These cell lines were cultured as described in a previous study.^15^

### 2.2 Knockdown and overexpression of Gal‐3 and CXCR2 by lentivirus‐mediated system

Lentivirus‐mediated silencing and overexpression of Gal‐3 and CXCR2 of the RCC cells were performed as described in a previous study.^15^ We obtained pLKO.1 plasmid containing shRNA targeting human Gal‐3 (shGal‐3#1, Clone ID TRCN0000029305; shGal‐3#2, Clone ID TRCN0000029307) and CXCR2 (shCXCR2, Clone ID TRCN0000009138) from the National RNAi Core Facility (Academia Sinica, Taipei, Taiwan). Full‐length DNA encoding Gal‐3 and CXCR2 genes were amplified using RT‐PCR and cloned to pLAS2w. The primer sequences for cloning the full length of Gal‐3 and CXCR2 are listed in Table S1. To knockdown Gal‐3 in RCC spheres, A‐498‐derived primary tumour spheres were dissociated into single cells, re‐seeded in a 10% FBS‐RPMI medium, infected with shGal‐3 or shLuc lentivirus, and then cultured to form secondary spheres (2S).

### 2.3 RT‐qPCR

RT‐qPCR was performed as described in a previous study.^15^ The specific primers used in the RT‐qPCR are presented in Table S2.

### 2.4 Western blotting

Total proteins were extracted using the RIPA Lysis buffer (Millipore, Temecula). Equal amounts (40 μg) were separated on 13% SDS‐PAGE, electro‐transferred onto a nitrocellulose membrane (Millipore), incubated with antibodies against the Nanog, Sox2, Oct4 (Cell Signaling), CXCR2, galectin‐3 (R&D Systems, Minneapolis, MN), **α‐**tubulin (Epitomics Inc, Burlingame, CA) and horseradish peroxidase (HRP)‐conjugated secondary antibodies and then analysed through enhanced chemiluminescence (ECL).

### 2.5 Drug sensitivity

A‐498 parental and sphere cells (1 × 10^3^) transduced with either shLuc or shGal‐3 were cultured in 96‐well microtitre plates with a total volume of 100 μL/well. After 16 hour, we treated the cells with various concentrations of sunitinib malate or sorafenib (Santa Cruz Biotechnology) for 72 hour and then examined cell viability using the CellTiter‐Glo Luminescent cell viability assay following the manufacturer's instructions (Promega, Madison, WI).

### 2.6 Cell migration and invasion assays

We evaluated tumour cell migration and invasion assays using transwell assay (Costar, 8‐μm pore; Corning, NY) as described in a previous study.^15^

### 2.7 Colony formation assay

RCC cells (1 × 10^3^) were suspended in 0.33% Bacto‐agar (Sigma‐Aldrich) and then layered over 0.5% Bacto‐agar in six‐well plates. On day 30, we counted the colonies after fixing them with methanol and staining them with Giemsa.

### 2.8 Sphere formation

RCC cells were cultured in a tumour sphere medium (Gibco, BRL, Life Technologies) that contained serum‐free DMEM/F12 (1:1) medium, 1X B27 supplement, 20 ng/mL human recombinant basic fibroblast growth factor (bFGF) and 20 ng/mL epidermal growth factor (EGF). RCC cells were seeded at 500 cells per 96‐well (or 8 × 10^4^ per 6‐well), and the tumour sphere medium was replaced with fresh medium every 3‐4 days. ACHN cells were cultured for 14 days, while A‐498 and Caki‐1 cells were cultured for 21 days. We used a microscope to count the spheres. Primary spheres (1° sphere) were dissociated to single cells and re‐seeded to yield the second generation (2° sphere).

### 2.9 In vivo tumour growth

We purchased male NOD/SCID mice (NOD.CB17‐Prkdcscid/IcrCrlBltw) from BioLASCO Taiwan Co., Ltd. and maintained them under specific pathogen‐free conditions at the Animal Center of National Yang‐Ming University, as approved by the university's Institutional Animal Care and Use Committee. To establish a xenograft tumour model, empty vector (ev)‐ or Gal‐3‐infected Caki‐1 (3 × 10^3^‐10^5^/100 μL) monolayer or sphere cells were subcutaneously implanted into the abdominal flanks of six‐ to eight‐week‐old male NOD/SCID mice. We measured tumour size with a calliper and calculated it as length × width × height (in mm^3^) every week.

### 2.10 Flow cytometry

Cells (1 × 10^6^) were incubated with anti‐CXCR2 monoclonal antibodies (R&D Systems) at 4°C for 1 hour and then stained with fluorescein isothiocyanate (FITC)‐anti‐mouse‐IgG at 4°C for 30 minutes, and analysed with flow cytometry (BD FACSCalibur).

### 2.11 Tissue microarray (TMA) and immunohistochemistry (IHC)

We purchased tissue microarray slides from Biomax (US Biomax Inc., Rockville, MD) and performed IHC as described in a previous study.^15^ TMA slides were incubated with the Gal‐3 or CXCR2 (R&D system, Minneapolis, MN) primary antibody. The final value was the sum of the percentage of the stained area and the intensity of the stained cells. The detailed clinicopathologic characteristics of the patients included in the TMA are listed in Table S3.

### 2.12 Statistical analysis

Data are expressed as the mean ± SD. Differences between two groups were determined using Student's *t* test. We adopted the SurvExpress^16^ web‐based tool to analyse the gene expression of Gal‐3 and CXCR2 in ccRCC (accession no. KIRC‐TCGA). Survival durations were analysed using the Kaplan‐Meier method and compared in the patient groups with the log‐rank test. Using Cox survival analysis, we classified a population of ccRCC patients into high‐risk and low‐risk groups in accordance with their prognostic index. Statistical significance was set at *P* < 0.05.

## 3 RESULTS

### 3.1 Enrichment of renal CSCs

**Figure 1:**
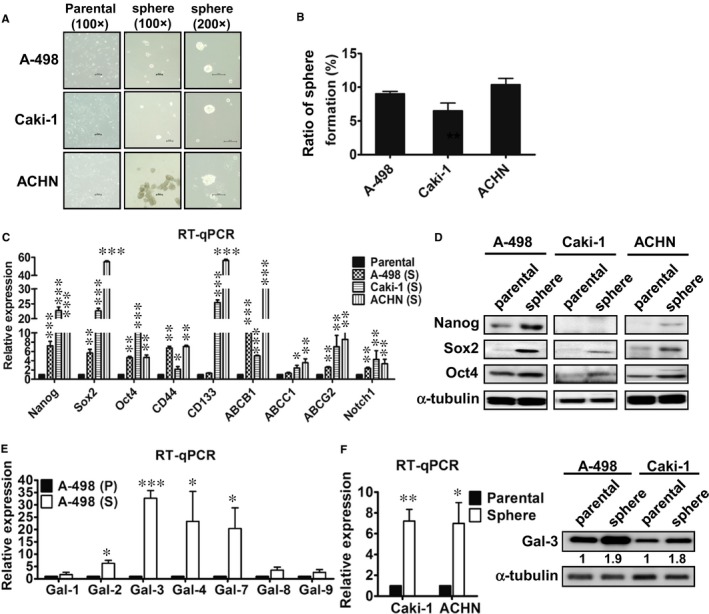
Enrichment of tumour spheres and galectin‐3 was highly expressed in the tumour spheres of renal cancer cell lines. (A) RCC cells were cultured in a defined serum‐free selection tumour sphere medium for 21 days. (B) The ratio of sphere formation (%) in the RCC cells was measured. (C) The mRNA levels of stemness‐related genes were evaluated in the parental and tumour spheres of kidney cancer cells using RT‐qPCR. (D) The protein levels of stemness‐related genes Nanog, Sox2 and Oct4 were analysed using western blotting. (E) The mRNA levels of the galectin family were detected in parental A‐498 (P) and A‐498 spheres (S) using RT‐qPCR. (F) The mRNA levels of galectin‐3 were also analysed in the parental and tumour spheres of Caki‐1 and ACHN cells. The protein levels of galectin‐3 (Gal‐3) in both the parental and spheres of A‐498 and Caki cells were analysed using Western blotting. The reported results are representative of three independent experiments. *P < 0.05, **P < 0.01, ***P < 0.001

To determine whether cultured human RCC cell lines contained a population of CSCs, RCC cells were cultured in a defined serum‐free selection tumour sphere medium for a few days. The morphology of the RCC cell spheres is shown in Figure 1A. We observed only 9% sphere formation in A‐498, 7% in Caki‐1 and 11% in ACHN cells (Figure 1B). The stemness‐associated genes were analysed using RT‐qPCR, and the results showed that the mRNA levels of Nanog, Sox2, Oct4, CD44, CD133, ABCB1, ABCC1, ABCG2 and Notch1 were significantly increased in RCC tumour spheres compared with parental cells (Figure 1C). Furthermore, we adopted Western blotting to confirm the protein levels of Nanog, Sox2 and Oct4 in three RCC tumour spheres (Figure 1D).

### 3.2 Galectin‐3 was highly expressed in the tumour spheres of RCC cells

Galectins have been reported to promote cancer cells’ chemoresistance and CSC formation.^12^, ^17^ Therefore, we analysed the galectin levels in renal CSCs using RT‐qPCR. Regarding the galectin family, the expression of Gal‐2, Gal‐3, Gal‐4 and Gal‐7 was significantly increased in A‐498 CSCs compared with parental cells. Of those, Gal‐3 demonstrated a more than 30‐fold increase in RCC tumour spheres (Figure 1E). We then utilized other RCC cells to verify whether Gal‐3 was also up‐regulated in these renal tumour spheres and found that Gal‐3 mRNA expression demonstrated a significant sevenfold increase in the tumour spheres of Caki‐1 and ACHN cells (Figure 1F). Western blotting was further adopted to confirm the Gal‐3 expression in RCC cells. Compared with parental cells, tumour spheres expressed levels of galectin‐3 protein that were twice as high (Figure 1F).

### 3.3 Knockdown of galectin‐3 in parental RCC cells decreased self‐renewal capacity and drug resistance

**Figure 2:**
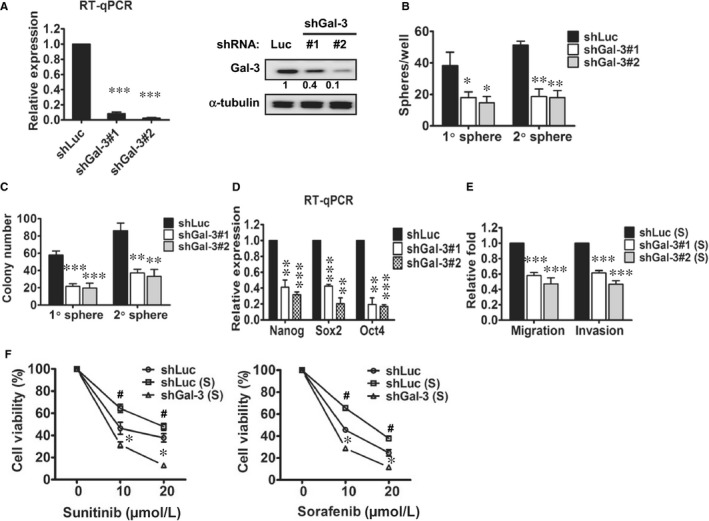
Knockdown of galectin‐3 in parental A‐498 cells decreased motility, self‐renewal capacity and drug resistance. (A) Galectin‐3 was suppressed using lentivirus delivery of shGal‐3 into A‐498 cells. The mRNA and protein levels of galectin‐3 were detected in the shGal‐3‐infected A‐498 cells (#1 and #2) compared with those in the shLuc‐infected cells (shLuc) using both RT‐qPCR and Western blotting. (B) Primary (1° sphere) and secondary sphere (2° sphere) formation were analysed in the shLuc‐ and shGal‐3‐infected A‐498 cells. Colony‐forming (C), stemness‐related gene expression levels (D), and invasiveness and migration (E) abilities were examined in the spheres of shLuc‐ and shGal‐3‐infected A‐498 cells. *P < 0.05, **P < 0.01, ***P < 0.001. (F) The drug sensitivities of shLuc‐ and shGal‐3‐infected A‐498 cell spheres were measured using sunitinib and sorafenib treatment. ^#^ *P* < 0.05: shLuc (S) compared with shLuc; *P < 0.05: shGal‐3 (S) compared with shLuc (S). The reported results are representative of three independent experiments

To determine the role of Gal‐3 in cell motility and the sphere‐forming ability of RCC cells, we used the lentivirus‐mediated delivery of galectin‐3 shRNA (shGal‐3) to knockdown Gal‐3 in A‐498 cells. Significantly decreased Gal‐3 expression was observed in cells infected with the shGal‐3 virus compared to cells infected with the shLuc virus with regard to both mRNA and protein levels (Figure 2A). As successful sphere formation of cancer stem cells is a key behaviour of CSCs for evaluating in vitro self‐renewal property, we were able to determine the sphere formation capacity of A‐498 cells through stable Gal‐3 knockdown. After suppressing Gal‐3, primary (1° sphere) and secondary sphere (2° sphere) formations were significantly reduced by 50%‐60% in RCC cells (Figure 2B). Furthermore, Gal‐3 silencing significantly inhibited the anchorage‐independent growth ability of sphere cells using colony formation assay (Figure 2C), while the mRNA levels of Nanog, Sox2 and Oct4 were significantly decreased in A‐498/shGal‐3 sphere cells (Figure 2D). Functionally, the knockdown of Gal‐3 significantly reduced cell invasion and migration in RCC sphere cells by 40%‐50% (Figure 2E). As the CSCs were the postulated mediators of drug resistance, we examined the drug sensitivity of Gal‐3‐silenced sphere cells. As shown in Figure 2F, sphere cells (shLuc (S)) were significantly more resistant to sunitinib and sorafenib compared to parental cells (shLuc). Furthermore, Gal‐3 knockdown sphere cells (shGal‐3 (S)) were significantly more sensitive to sunitinib and sorafenib treatment compared to control sphere cells (shLuc (S)).

### 3.4 Galectin‐3 maintained the stemness properties of renal CSCs

**Figure 3:**
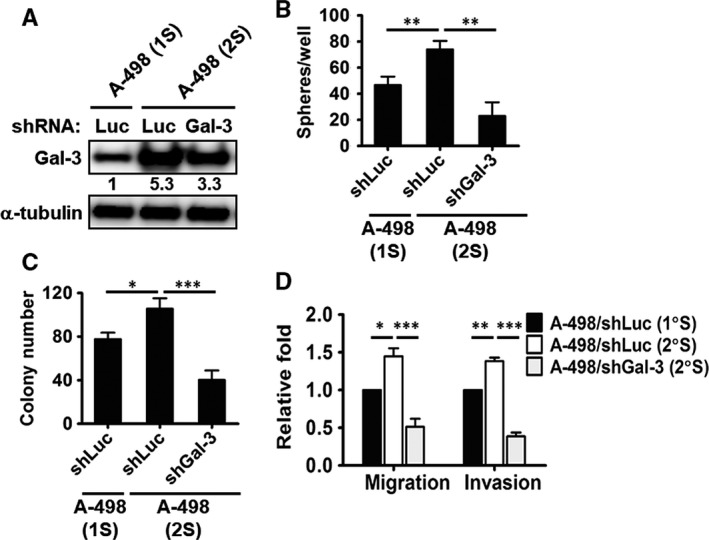
Galectin‐3 maintained the stemness properties of renal CSCs. (A) A‐498 primary tumour spheres (1S) were infected with shGal‐3 lentivirus and then cultured to form spheres (2S). Galectin‐3 proteins were examined in shLuc‐ and shGal‐3‐infected sphere cells. Sphere‐forming (B), colony‐forming (C), and invasion and migration (D) abilities were analysed in the shLuc‐ and shGal‐3‐infected A‐498 sphere cells. The reported results are representative of three independent experiments. *P < 0.05, **P < 0.01, ***P < 0.001

To further examine the role of Gal‐3 in maintaining CSCs, we infected A‐498 primary tumour spheres with shGal‐3 lentivirus and then cultured them to form secondary spheres. First, we used Western blot to confirm galectin‐3 knockdown in secondary spheres (Figure 3A). Furthermore, silencing Gal‐3 significantly reduced the sphere formation (Figure 3B), anchorage‐independent growth (Figure 3C), migration and invasion (Figure 3D) ability of the secondary sphere cells. Therefore, we also observed Gal‐3 to participate in the maintenance of the stemness features of renal CSCs.

### 3.5 Down‐regulation of chemokine/cytokine expression in Gal‐3‐knockdown RCC tumour spheres

**Figure 4:**
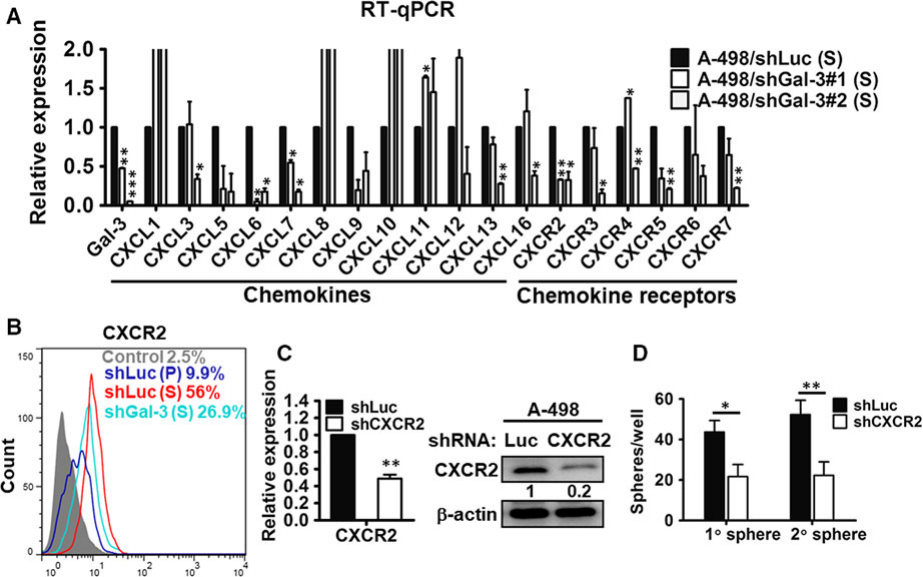
Down‐regulation of chemokine expression in galectin‐3‐knockdown A‐498 tumour spheres. (A) We screened the mRNA expression levels of chemokines and chemokine receptors in the tumour spheres of shLuc‐ and shGal‐3‐infected A‐498 using RT‐qPCR. (B) CXCR2 levels in parental (P) and sphere (S) cells were examined with flow cytometry. (C) Knockdown of CXCR2 in A‐498 cells was confirmed with RT‐qPCR and Western blotting. (D) The sphere‐forming abilities of shLuc‐ and shCXCR2‐infected A‐498 cells. The reported results are representative of three independent experiments. *P < 0.05, **P < 0.01, ***P < 0.001

To study the molecular mechanism of Gal‐3 in the CSCs, we analysed chemokine and chemokine receptor levels using RT‐qPCR. As shown in Figure 4A, the expressions of several chemokines were reduced in both clones of the shGal‐3‐infected renal CSCs, including CXCL5, CXCL6, CXCL7 and CXCL9. The chemokine receptors CXCR2 and CXCR5 were also significantly down‐regulated in the Gal‐3 silenced CSCs. As CXCR2 and its ligands (CXCL6 and CXCL7) were simultaneously inhibited in shGal‐3 CSCs, we focused on the role of CXCR2 in the Gal‐3‐mediated formation of renal CSCs and further confirmed CXCR2 expression in shGal‐3 CSCs by flow cytometry (Figure 4B). Compared with the parental A‐498 cells (9.9%), the sphere cells expressed more CXCR2 (56%), and Gal‐3 silencing decreased CXCR2 expression in the CSCs (26.9%).

### 3.6 Suppression of CXCR2 led to decreased sphere‐forming ability in RCC cells

We silenced CXCR2 in parental A‐498 cells to investigate the role of CXCR2 in CSC formation. Compared to cells infected with the control virus expressing shLuc, cells infected with the shCXCR2 virus expressed lower levels of this particular chemokine receptor (Figure 4C). Furthermore, sphere‐forming ability was significantly down‐regulated by 50%‐60% in the shCXCR2‐infected RCC cells (Figure 4D).

### 3.7 Overexpression of CXCR2 in shGal‐3‐infected RCC cells restored cell motility, colony formation and self‐renewal capacity

**Figure 5:**
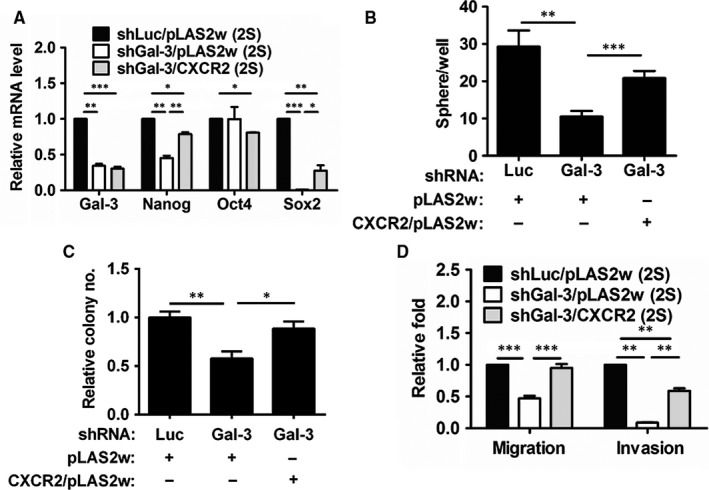
Overexpression of CXCR2 in shGal‐3‐infected A‐498 cells recovered cell motility and self‐renewal capacity. (A) Gene expression in shLuc‐ (shLuc/pLAS2w), shGal‐3‐ (shGal‐3/pLAS2w), and shGal‐3‐infected/lentivirus‐mediated overexpressed CXCR2 (shGal‐3/CXCR2) A‐498 cells were detected by RT‐qPCR. Sphere‐forming (B), colony‐forming (C), and migration and invasion (D) abilities were examined in secondary sphere cells. The reported results are representative of three independent experiments. *P < 0.05, **P < 0.01, ***P < 0.001

To explore the role of CXCR2 in the formation of Gal‐3‐mediated CSCs, we infected Gal‐3 knockdown (shGal‐3) A‐498 cells with lentivirus carrying the CXCR2 gene (pLAS2w/CXCR2). Compared to cells infected with the shLuc control virus, Gal‐3 silencing significantly decreased the Nanog and Sox2 expression (Figure 5A), sphere formation (Figure 5B), anchorage‐independent growth (Figure 5C) and motility (Figure 5D) of RCC sphere cells. In contrast, CXCR2 overexpression significantly restored the expression of the Nanog and Sox2 expression (Figure 5A), sphere formation (Figure 5B), colony formation (Figure 5C) and motility (Figure 5D) of shGal‐3 RCC sphere cells.

### 3.8 Overexpression of galectin‐3 in RCC cells promoted sphere‐forming capacity and in vitro and in vivo tumorigenicity

**Figure 6:**
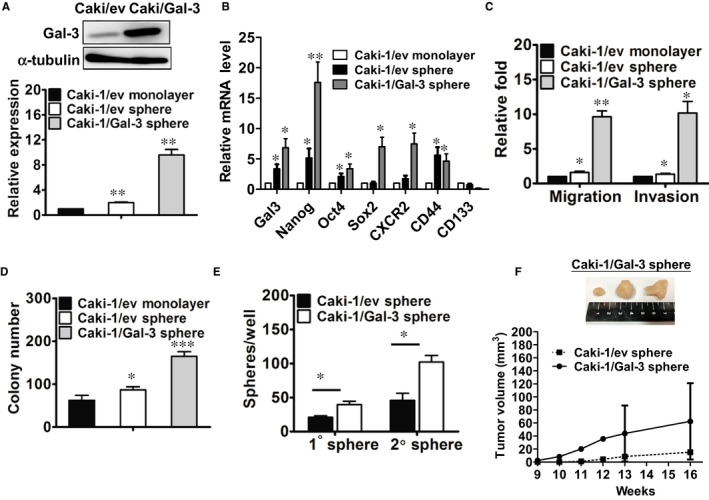
Overexpression of galectin‐3 in Caki‐1 cells promoted sphere‐forming capacity and in vivo tumorigenicity. Galectin‐3 (A) and stemness‐related gene (B) expressions in monolayer and spheres of empty vector (ev)‐infected and Gal‐3‐infected Caki‐1 cells were analysed using Western blot and RT‐qPCR. Migration and invasion (C), colony‐forming (D) and sphere formation (E) abilities were all examined. The reported results are representative of three independent experiments. *P < 0.05, **P < 0.01, ***P < 0.001. (F) Empty vector (ev)‐ or Gal‐3‐infected Caki‐1 (3 × 10^5^/100 μL) sphere cells were subcutaneously implanted into the abdominal flanks of NOD/SCID nude mice (n = 4). We measured tumour size with a calliper and calculated it as length × width × height (in mm^3^) every week

We used lentivirus‐mediated Gal‐3 overexpression in Caki‐1 cells to further confirm the role of Gal‐3 in renal CSC formation. First, Gal‐3 overexpression in RCC cells was confirmed by RT‐qPCR and western blot (Figure 6A). Compared to parental RCC cells, sphere cells with an empty vector expressed higher levels of galectin‐3, Oct4, Nanog, Sox2 and CD44 (Figure 6B). Overexpression of Gal‐3 considerably promoted these stemness genes and CXCR2 expression in RCC sphere cells (Figure 6B). Furthermore, migration, invasion (Figure 6C), colony formation (Figure 6D) and sphere‐forming ability (Figure 6E) were all significantly up‐regulated in Gal‐3‐infected RCC sphere cells. To assess the tumour growth capacity of renal cancer stem cells, we subcutaneously injected 3 × 10^5^ of Caki‐derived monolayers or tumour spheres into NOD/SCID mice (n = 4). In contrast to no tumour formation in the monolayer group and one mouse with tumour formation in the RCC sphere group, the Gal‐3‐overexpressed RCC sphere cells generated tumour formation in three of four mice (data not shown). Furthermore, tumours generated by the Gal‐3‐overexpressed RCC spheres were larger than the RCC sphere‐derived tumours (Figure 6F). These results suggest that Gal‐3 overexpression in RCC sphere cells promote in vivo tumour growth.

### 3.9 Galectin‐3 expression correlated with CXCR2, tumour progression and prognosis in RCC tissues

**Figure 7:**
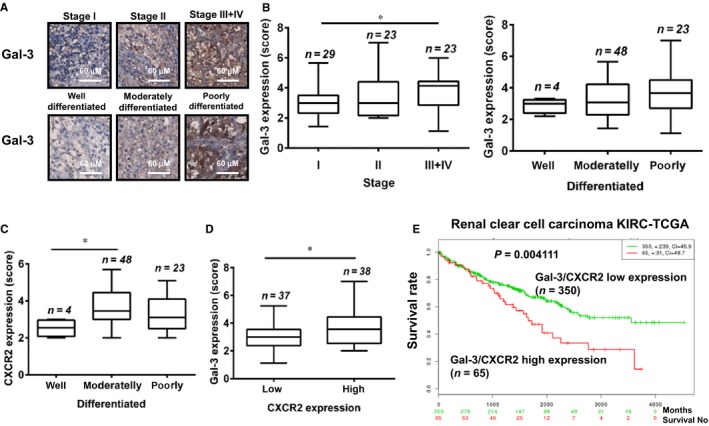
Galectin‐3 expression correlated with CXCR2, tumour progression and prognosis in the tissues of kidney cancer. (A‐D) We performed IHC staining using anti‐galectin‐3 and anti‐CXCR2 antibodies on tissue microarrays that contained samples from 75 patients with ccRCC. (A, B) Galectin‐3 expression levels were quantified in various stages and differentiated states of ccRCC. (C) CXCR2 expression levels were quantified in differentiated states of ccRCC. (D) Patients were divided into two groups (low and high) based on whether their CXCR2 levels were below or above the median value. Galectin‐3 expression levels were compared between low and high levels of CXCR2 in ccRCC tissues. *P < 0.05. (E) Kaplan‐Meier curves according to galectin‐3 and CXCR2 expression levels in patients with ccRCC were conducted using SurvExpress web‐based analysis. Censoring samples are shown as “+” marks. Data set, concordance index (CI) and *P*‐value of the log‐rank test are shown. Red and green curves denoted high‐risk (high expression levels) and low‐risk (low expression levels) groups, respectively

To investigate the expression of Gal‐3 and CXCR2 in human RCC tissues, we performed immunohistochemistry staining on tissue microarrays that contained samples from 75 patients with ccRCC. We observed higher Gal‐3 expression in the advanced stages (III+IV) and grade (poorly differentiated) RCC tissues (Figure 7A and B). CXCR2 expression was significantly correlated with tumour differentiation (Figure 7C) but not RCC stage (data not shown). Furthermore, Gal‐3 expression was significantly higher in the CXCR2 high expression group than in the low expression group (Figure 7D). To further study the correlation between the expression levels of Gal‐3/CXCR2 and patient prognosis, we used the online tool SurvExpress^16^ to analyse 415 patients with various stages of ccRCC. Using Cox survival analysis, we found that patients with higher co‐expressions of Gal‐3 and CXCR2 had a significantly worse survival rate (Figure 7E) and that Gal‐3 expression correlated with CXCR2 expression, tumour progression and prognosis in RCC.

## 4 DISCUSSION

The overexpression of Gal‐3 is associated with the increased invasiveness of many kinds of tumours. Higher levels of Gal‐3 are found in the sera of cancer patients with metastasis.^18^ Gal‐3 promotes cancer progression through intra‐ and extra‐cellular mechanisms in the tumour microenvironment. Intracellular Gal‐3 interacts with RAS and β‐catenin to enhance cell transformation and proliferation.^8^, ^9^, ^10^ Furthermore, Gal‐3 augments tumour stem cell property and drug resistance through its interaction with β‐catenin.^12^ Several chemokine and chemokine receptor genes, such as CXCR4, CXCR7 and CCL5,^19^, ^20^ are the downstream genes of β‐catenin. In this study, we found that Gal‐3 overexpression may promote CXCR2 to augment the stemness property of RCC. In our previous study, cancer spheres secreted higher levels of Gal‐3, while Gal‐3 knockdown reduced secretion levels. Recombinant Gal‐3 promotes cancer sphere formation.^12^ Furthermore, previous studies have demonstrated Gal‐3 to interact with epidermal growth factor receptor (EGFR) and transforming growth factor‐β receptor (TGFβR).^9^ Therefore, extracellular Gal‐3 may also stimulate sphere formation in collaboration with EGF or bFGF signalling in the tumour sphere medium.

Existing evidence indicates that drug resistance regulation by Gal‐3 may result from intracellular effects on the apoptotic pathways.^11^ The anti‐apoptotic mechanisms of Gal‐3 include: (1) the phosphorylation status of Gal‐3,^21^ (2) the Gal‐3 translocation from the nucleus to the cytoplasm,^22^ (3) the regulation of mitochondrial membrane potential,^23^ (4) the modulation of survival signalling pathway,^24^ and (5) the regulation of the caspase pathway.^25^ Furthermore, Gal‐3 plays a crucial role in regulating the Wnt/β‐catenin signalling pathway. The best evidence so far of the importance of the Wnt pathway to CSCs biology has been reported in myeloid leukaemia, but its contribution has also been reported in the maintenance of the CSCs of melanoma, breast, colon, and lung cancers.^12^, ^26^ Therefore, with regard to inhibiting the common upstream regulator of Wnt/β‐catenin signalling, Gal‐3 may be an effective target for cancer stem cell therapy.

Clear evidence has shown that CXCR2 and its associated ligands play important roles in various types of cancer. Most of the ELR^+^ CXC chemokines that have been described as promoters of tumour angiogenesis are CXCR2 ligands, namely CXCL1, 2, 3, 5, 6, 7 and 8.^27^ In addition to the role in angiogenesis, CXCR2 ligands have also been implicated in the processes of tumour growth and neutrophil recruitment to the site of the tumour.^27^ CXCL1 is overexpressed in colorectal tumours, and blocking CXCL1 in vivo with antibodies can combat tumour growth enhancement by PGE2.^28^ CXCL5 promotes prostate cancer cell proliferation and invasion,^29^ while CXCL5 antibodies reduce lung tumour growth in mice.^30^ IL‐8 (CXCL8) expression correlates with tumour growth and poor outcome in various cancers,^31^, ^32^ while IL‐8 antibodies can restrict colorectal cancer growth in vivo.^33^ CXCL7 is an independent prognostic factor for the overall survival rate of RCC, while CXCL7 antibodies reduce tumour growth in nude mice.^34^

CXCR2 has been observed to participate in primary brain tumour growth.^35^ In a mammary gland tumour model, macrophages increase the expression of inflammatory chemokines and promote tumour cell invasion by activating CXCR2.^36^ CXCR2 knockdown promotes the chemo‐sensitivity of breast cancer cells and prevents tumour growth, angiogenesis and lung metastasis.^37^ Furthermore, a CXCR2 blockade reduces tumorigenesis in lung, oesophageal, pancreatic and kidney cancers.^3^, ^38^, ^39^, ^40^ However, the introduction of CXCR2 and its ligands by K‐RAS reinforces senescence in vitro.^41^ CXCR2 inhibition but not germline knockout of CXCR2 can slow tumorigenesis in the pancreas.^40^ Therefore, CXCR2 and its ligands are thought to serve a protective function in the early stages of tumorigenesis, thus complicating the role of CXCR2 in cancer formation and progression.

In the plasma of RCC patients, CXCR2 ligands CXCL1, CXCL3, CXCL5 and CXCL8 are elevated chemokines.^42^ Furthermore, CXCL7 is a prognostic factor for the overall survival of RCC. CXCL7 promotes RCC cell proliferation both in vitro and in vivo, and a CXCL7/CXCR2 blockade by antibody or inhibitor reduces tumour growth in mice.^34^ Altogether, these findings indicate the importance of CXCR2 in the progression and targeting therapy of RCC.^43^

In summary, we have demonstrated that highly expressed Gal‐3 can up‐regulate CXCR2 to augment the stemness property of RCC. Gal‐3 and CXCR2 expressions were correlated with RCC tumour progression, and Gal‐3 expression correlated with CXCR2 expression in RCC tissues. As we found that higher co‐expressions of Gal‐3 and CXCR2 correlated with a significantly worse survival rate, Gal‐3 may be a prognostic biomarker and innovative target for the combined modality therapy of RCC.

## CONFLICT OF INTEREST

All authors declare to have no conflicts of interest with regard to this study.

## References

[1] Ljungberg B , Bensalah K , Canfield S , et al. EAU guidelines on renal cell carcinoma: 2014 update. Eur Urol. 2015;67:913‐924. 25616710 10.1016/j.eururo.2015.01.005

[2] Siegel RL , Miller KD , Jemal A . Cancer statistics, 2016. CA Cancer J Clin. 2016;66:7‐30. 26742998 10.3322/caac.21332

[3] Beck B , Blanpain C . Unravelling cancer stem cell potential. Nat Rev Cancer. 2013;13:727‐738. 24060864 10.1038/nrc3597

[4] Pattabiraman DR , Weinberg RA . Tackling the cancer stem cells‐ what challenges do they pose? Nat Rev Drug Discov. 2014;13:497‐512. 24981363 10.1038/nrd4253 PMC4234172

[5] Kise K , Kinugasa‐Katayama Y , Takakura N . Tumor microenvironment for cancer stem cells. Adv Drug Deliv Rev. 2016;99(Pt B):197‐205. 26362921 10.1016/j.addr.2015.08.005

[6] Ruvolo PP . Galectin 3 as a guardian of the tumor microenvironment. Biochim Biophys Acta. 2016;1863:427‐437. 26264495 10.1016/j.bbamcr.2015.08.008

[7] Yang RY , Rabinovich GA , Liu FT . Galectins: structure, function and therapeutic potential. Expert Rev Mol Med. 2008;10:e17. 18549522 10.1017/S1462399408000719

[8] Shalom‐Feuerstein R , Plowman SJ , Rotblat B , et al. K‐ras nanoclustering is subverted by overexpression of the scaffold protein galectin‐3. Cancer Res. 2008;68:6608‐6616. 18701484 10.1158/0008-5472.CAN-08-1117 PMC2587079

[9] Newlaczyl AU , Yu LG . Galectin‐3–a jack‐of‐all‐trades in cancer. Cancer Lett. 2011;313:123‐128. 21974805 10.1016/j.canlet.2011.09.003

[10] Shimura T , Takenaka Y , Tsutsumi S , et al. Galectin‐3, a novel binding partner of beta‐catenin. Cancer Res. 2004;64:6363‐6367. 15374939 10.1158/0008-5472.CAN-04-1816

[11] Fukumori T , Kanayama HO , Raz A . The role of galectin‐3 in cancer drug resistance. Drug Resist Updat. 2007;10:101‐108. 17544840 10.1016/j.drup.2007.04.001 PMC3626271

[12] Chung LY , Tang SJ , Wu YC , et al. Galectin‐3 augments tumor initiating property and tumorigenicity of lung cancer through interaction with β‐catenin. Oncotarget. 2015;6:4936‐4952. 25669973 10.18632/oncotarget.3210 PMC4467125

[13] Nangia‐Makker P , Honjo Y , Sarvis R , et al. Galectin‐3 induces endothelial cell morphogenesis and angiogenesis. Am J Pathol. 2000;156:899‐909. 10702407 10.1016/S0002-9440(10)64959-0 PMC1876842

[14] Sakaki M , Fukumori T , Fukawa T , et al. Clinical significance of Galectin‐3 in clear cell renal cell carcinoma. J Med Invest. 2010;57:152‐157. 20299755 10.2152/jmi.57.152

[15] Huang CS , Tang SJ , Chung LY , et al. Galectin‐1 upregulates CXCR4 to promote tumor progression and poor outcome in kidney cancer. J Am Soc Nephrol. 2014;25:1486‐1495. 24511119 10.1681/ASN.2013070773 PMC4073433

[16] Aguirre‐Gamboa R , Gomez‐Rueda H , Martínez‐Ledesma E , et al. SurvExpress: an online biomarker validation tool and database for cancer gene expression data using survival analysis. PLoS ONE. 2013;8:e74250. 24066126 10.1371/journal.pone.0074250 PMC3774754

[17] Chung LY , Tang SJ , Sun GH , et al. Galectin‐1 promotes lung cancer progression and chemoresistance by upregulating p38 MAPK, ERK, and cyclooxygenase‐2. Clin Cancer Res. 2012;18:4037‐4047. 22696230 10.1158/1078-0432.CCR-11-3348

[18] Iurisci I , Tinari N , Natoli C , Angelucci D , Cianchetti E , Iacobelli S . Concentrations of galectin‐3 in the sera of normal controls and cancer patients. Clin Cancer Res. 2000;6:1389‐1393. 10778968

[19] Aman A , Piotrowski T . Wnt/beta‐catenin and Fgf signaling control collective cell migration by restricting chemokine receptor expression. Dev Cell. 2008;15:749‐761. 19000839 10.1016/j.devcel.2008.10.002

[20] Yasuhara R , Irié T , Suzuki K , et al. The β‐catenin signaling pathway induces aggressive potential in breast cancer by up‐regulating the chemokine CCL5. Exp Cell Res. 2015;338:22‐31. 26363360 10.1016/j.yexcr.2015.09.003

[21] Yoshii T , Fukumori T , Honjo Y , et al. Galectin‐3 phosphorylation is required for its anti‐apoptotic function and cell cycle arrest. J Biol Chem. 2002;277:6852‐6857. 11724777 10.1074/jbc.M107668200

[22] Takenaka Y , Fukumori T , Yoshii T , et al. Nuclear export of phosphorylated galectin‐3 regulates its antiapoptotic activity in response to chemotherapeutic drugs. Mol Cell Biol. 2004;24:4395‐4406. 15121858 10.1128/MCB.24.10.4395-4406.2004 PMC400475

[23] Fukumori T , Oka N , Takenaka Y , et al. Galectin‐3 regulates mitochondrial stability and antiapoptotic function in response to anticancer drug in prostate cancer. Cancer Res. 2006;66:3114‐3119. 16540661 10.1158/0008-5472.CAN-05-3750

[24] Oka N , Nakahara S , Takenaka Y , et al. Galectin‐3 inhibits tumor necrosis factor‐related apoptosis‐inducing ligand‐induced apoptosis by activating Akt in human bladder carcinoma cells. Cancer Res. 2005;65:7546‐7553. 16140916 10.1158/0008-5472.CAN-05-1197

[25] Yu F , Finley RL , Raz A , et al. Galectin‐3 translocates to the perinuclear membranes and inhibits cytochrome c release from the mitochondria. A role for synexin in galectin‐3 translocation. J Biol Chem. 2002;277:15819‐15827. 11839755 10.1074/jbc.M200154200

[26] Hu Y , Fu L . Targeting cancer stem cells: a new therapy to cure cancer patients. Am J Cancer Res. 2012;2:340‐356. 22679565 PMC3365812

[27] Mukaida N , Sasaki S , Baba T . Chemokines in cancer development and progression and their potential as targeting molecules for cancer treatment. Mediators Inflamm. 2014;2014:170381. 24966464 10.1155/2014/170381 PMC4055660

[28] Wang D , Wang H , Brown J , et al. CXCL1 induced by prostaglandin E2 promotes angiogenesis in colorectal cancer. J Exp Med. 2006;203:941‐951. 16567391 10.1084/jem.20052124 PMC2118273

[29] Begley LA , Kasina S , Mehra R , et al. CXCL5 promotes prostate cancer progression. Neoplasia. 2008;10:244‐254. 18320069 10.1593/neo.07976 PMC2262133

[30] Arenberg DA , Keane MP , DiGiovine B , et al. Epithelial‐neutrophil activating peptide (ENA‐78) is an important angiogenic factor in non‐small cell lung cancer. J Clin Invest. 1998;102:465‐472. 9691082 10.1172/JCI3145 PMC508906

[31] Horikawa T , Kaizaki Y , Kato H , et al. Expression of interleukin‐8 receptor A predicts poor outcome in patients with nasopharyngeal carcinoma. Laryngoscope. 2005;115:62‐67. 15630368 10.1097/01.mlg.0000150675.37860.f7

[32] Murphy C , McGurk M , Pettigrew J , et al. Nonapical and cytoplasmic expression of interleukin‐8, CXCR1, and CXCR2 correlates with cell proliferation and microvessel density in prostate cancer. Clin Cancer Res. 2005;11:4117‐4127. 15930347 10.1158/1078-0432.CCR-04-1518

[33] Hwang WL , Yang MH , Tsai ML , et al. SNAIL regulates interleukin‐8 expression, stem cell‐like activity, and tumorigenicity of human colorectal carcinoma cells. Gastroenterology. 2011;141:279‐291. 21640118 10.1053/j.gastro.2011.04.008

[34] Grepin R , Guyot M , Giuliano S , et al. The CXCL7/CXCR1/2 axis is a key driver in the growth of clear cell renal cell carcinoma. Cancer Res. 2014;74:873‐883. 24335961 10.1158/0008-5472.CAN-13-1267

[35] Robinson S , Cohen M , Prayson R , et al. Constitutive expression of growth‐related oncogene and its receptor in oligodendrogliomas. Neurosurgery. 2001;48:864‐873. 11322447 10.1097/00006123-200104000-00035

[36] Bohrer LR , Schwertfeger KL . Macrophages promote fibroblast growth factor receptor‐driven tumor cell migration and invasion in a CXCR2‐dependent manner. Mol Cancer Res. 2012;10:1294‐1305. 22893608 10.1158/1541-7786.MCR-12-0275 PMC3553584

[37] Sharma B , Nawandar DM , Nannuru KC , et al. Targeting CXCR2 enhances chemotherapeutic response, inhibits mammary tumor growth, angiogenesis and lung metastasis. Mol Cancer Ther. 2013;12:799‐808. 23468530 10.1158/1535-7163.MCT-12-0529 PMC3653628

[38] Keane MP , Belperio JA , Xue YY , et al. Depletion of CXCR2 inhibits tumor growth and angiogenesis in a murine model of lung cancer. J Immunol. 2004;172:2853‐2860. 14978086 10.4049/jimmunol.172.5.2853

[39] Wu K , Cui L , Yang Y , et al. Silencing of CXCR2 and CXCR7 protects against esophageal cancer. Am J Transl Res. 2016;8:3398‐3408. 27648130 PMC5009392

[40] Steele CW , Karim SA , Leach JD , et al. CXCR2 inhibition profoundly suppresses metastases and augments immunotherapy in pancreatic ductal adenocarcinoma. Cancer Cell. 2016;29:832‐845. 27265504 10.1016/j.ccell.2016.04.014 PMC4912354

[41] Acosta JC , O'Loghlen A , Banito A , et al. Control of senescence by CXCR2 and its ligands. Cell Cycle. 2008;7:2956‐2959. 18838863 10.4161/cc.7.19.6780

[42] Mestas J , Burdick MD , Reckamp K , et al. The role of CXCR2/CXCR2 ligand biological axis in renal cell carcinoma. J Immunol. 2005;175:5351‐5357. 16210641 10.4049/jimmunol.175.8.5351

[43] Giuliano S , Guyot M , Grépin R , et al. The ELR+CXCL chemokines and their receptors CXCR1/CXCR2: a signaling axis and new target for the treatment of renal cell carcinoma. Oncoimmunology. 2014;3:e28399. 25050209 10.4161/onci.28399 PMC4063157

