# Supplementary material for: Galectin‐3 promotes CXCR2 to augment the stem‐like property of renal cell carcinoma
Source: J Cell Mol Med. 2018 Sep 24;22(12):5909–18. doi: 10.1111/jcmm.13860 (PMC6237593; doi:10.1111/jcmm.13860)
Supplement: Supplementary file 1 [file JCMM-22-5909-s001.docx]

**Supplementary Table 1.** Primer sequences used in cloning

| **Genes** | **Forward primer/Reverse primer** |
| --- | --- |
| galectin-3 | 5’- GCTAGCATGGCAGACAATTTTTCGC -3’  5’- GAATTCTTATATCATGGTATATGAAGC -3’ |
| CXCR2 | 5’- GGGCTAGCATGGAAGATTTTAACATGGAG -3’  5’- CCGAATTCTTAGAGAGTAGTGGAAGTGTG -3’ |

**Supplementary Table 2.** Primer sequences used in RT-qPCR

| **Genes** | **Forward primer/Reverse primer** |
| --- | --- |
| galectin-1 | 5’-GCCTGCCCGGGAACAT-3’  5’-CTGGCGACCAGACCACAAG-3’ |
| galectin-2 | 5’-GCTTCAGCGAATCCACCATT-3’  5’-GTTCTTGCCCCCAGTTGCT-3’ |
| galectin-3 | 5’-CCATTTGAAAGTGGGAAACCA-3’  5’-CATCATTCACTGCAACCTTGAAG-3’ |
| galectin-4 | 5’-CCAGCACCTCTTTGACTTTGC-3’  5’-CAATGTGTCCACCCTCTGGAA-3’ |
| galectin-7 | 5’-CTGGCACGGTGCTGAGAAT-3’  5’-GGAACCTGCTGGCATTGG-3’ |
| galectin-8 | 5’-TGAATGCAAATGCCAAAAGC-3’  5’-TGGGTTCAAGTGTAGAGCAATATCC-3’ |
| galectin-9 | 5’-ATGCTGTGGTCCGCAACA-3’  5’-GGCAGACTTCGCTCCTCAGA-3’ |
| Nanog | 5’-CCCAAAGGCAAACAACCCACTTCT-3’  5’-AGCTGGGTGGAAGAGAACACAGTT-3’ |
| Sox2 | 5’-GCACATGAACGGCTGGAGCAACG-3’  5’-TGCTGCGAGTAGGACATGCTGTAGG-3’ |
| Oct4 | 5’-GTGGAGGAAGCTGACAACAA-3’  5’-ATTCTCCAGGTTGCCTCTCA-3’ |
| CD44 | 5’-CCGCTATGTCCAGAAAGGA-3’  5’-CTGTCTGTGCTGTCGGTGAT-3’ |
| CD133 | 5’-TGGATGCAGAACTTGACAACGT-3’  5’-ATACCTGCTACGACAGTCGTGGT-3’ |
| ABCB1 | 5’-GCTCATCGTTTGTCTACAGTTCGT-3’  5’-ACAATGACTCCATCATCGAAACC-3’ |
| ABCC1 | 5’-TGCTGCACCAGTACTTCCACAT-3’  5’-CCCCAATGACAGCGGTCTT-3’ |
| ABCG2 | 5’-GCAGCTCTTCGGCTTGCA-3’  5’-CCCTGTTAATCCGTTCGTTTTT-3’ |
| Notch1 | 5’-CCTGAGGGCTTCAAAGTGTC-3’  5’-CGGAACTTCTTGGTCTCCAG-3’ |
| CXCL1 | 5’-AATCCTGCATCCCCCATA-3’  5’-TCAGTTGGATTTGTCACTG-3’ |
| CXCL3 | 5’-TGGTCACTGAACTGCGCT-3’  5’-ATGCGGGGTTGAGACAAG-3’ |
| CXCL5 | 5’-AGAGCTGCGTTGCGTTTGT-3’  5’-CTATGGCGAACACTTGCAGATTAC-3’ |
| CXCL6 | 5’-AGAGCTGCGTTGCACTTGTT-3’  5’-GCAGTTTACCAATCGTTTTGGGG-3’ |
| CXCL7 | 5’-GAACTCCGCTGCATGTGTATAAAG-3’  5’-GCATCTGGGTCCAGGCAG-3’ |
| CXCL8 | 5’-ACTGAGAGTGATTGAGAGTGGACC-3’  5’-ACAACCCTCTGCACCCAGTT-3’ |
| CXCL9 | 5’-CCAGTAGTGAGAAAGGGTCGC-3’  5’-AGGGCTTGGGGCAAATTGTT-3’ |
| CXCL10 | 5’-AAGCCAATTTTGTCCACGTGTT-3’  5’-TGGCCTTCGATTCTGGATTC-3’ |
| CXCL11 | 5’-GACGCTGTCTTTGCATAGGC-3’  5’-GGATTTAGGCATCGTTGTCCTTT-3’ |
| CXCL12 | 5’-TCTGAGAGCTCGCTTGAGTG-3’  5’-TCAGTTTCAGCAATGGTTT-3’ |
| CXCL13 | 5’-GCTTGAGGTGTAGATGTGTCC-3’  5’-CCCACGGGGCAAGATTTGAA-3’ |
| CXCL16 | 5’-GGCCCACCAGAAGCATTTAC-3’  5’-CTGAAGATGCCCCCTCTGAG-3’ |
| CXCR2 | 5’-CATGGCTTGATCAGCAAGGA-3’  5’-TGGAAGTGTGCCCTGAAGAAG-3’ |
| CXCR3 | 5’-CAGGTGCCCTCTTCAACATCA-3’  5’-ATGTTCAGGTAGCGGTCAAAGC-3’ |
| CXCR4 | 5’-CGTCAGTGAGGCAGATGAC-3’  5’-TGCAATAGCAGGACAGGATG-3’ |
| CXCR5 | 5’-GGTCACCCTACCACATCGTC-3’  5’-GCCATTCAGCTTGCAGGTATTG-3’ |
| CXCR6 | 5’-GACTATGGGTTCAGCAGTTTCA-3’  5’-GGCTCTGCAACTTATGGTAGAAG-3’ |
| CXCR7 | 5’-GGCTATGACACGCACTGCTACA-3’  5’-TGGTTGTGCTGCAC-3’ |
| GAPDH | 5’-CAACTACATGGTTTACATGTTC-3’  5’-GCCAGTGGACTCCACGAC-3’ |

**Supplementary Table 3.** Clinicopathologic characteristics of patients with kidney cancer included in this study

| **Case** | **Sex** | **Age** | **Organ** | **Pathology** | **Grade** | **Stage** | **TNM** |
| --- | --- | --- | --- | --- | --- | --- | --- |
| 1 | M | 34 | Kidney | clear cell carcinoma | G1-G2 | I | T1aN0M0 |
| 2 | M | 51 | Kidney | clear cell carcinoma | G1-G3 | I | T1aN0M0 |
| 3 | F | 56 | Kidney | clear cell carcinoma | G1-G2 | I | T1aN0M0 |
| 4 | F | 55 | Kidney | clear cell carcinoma | G2 | I | T1aN0M0 |
| 5 | M | 64 | Kidney | clear cell carcinoma | G2-G3 | I | T1aN0M0 |
| 6 | M | 47 | Kidney | clear cell carcinoma | G1-G2 | I | T1aN0M0 |
| 7 | M | 49 | Kidney | clear cell carcinoma | G2 | I | T1aN0M0 |
| 8 | M | 51 | Kidney | clear cell carcinoma | G1-G2 | I | T1aN0M0 |
| 9 | F | 57 | Kidney | clear cell carcinoma | G2 | I | T1aN0M0 |
| 10 | F | 40 | Kidney | clear cell carcinoma | G1 | I | T1aN0M0 |
| 11 | M | 64 | Kidney | clear cell carcinoma | G3 | I | T1aN0M0 |
| 12 | M | 64 | Kidney | clear cell carcinoma | G2 | I | T1bN0M0 |
| 13 | F | 61 | Kidney | clear cell carcinoma | G1 | I | T1aN0M0 |
| 14 | M | 44 | Kidney | clear cell carcinoma | G1 | I | T1aN0M0 |
| 15 | M | 41 | Kidney | clear cell carcinoma | G1-G2 | I | T1aN0M0 |
| 16 | M | 37 | Kidney | clear cell carcinoma | G1 | I | T1aN0M0 |
| 17 | F | 72 | Kidney | clear cell carcinoma | G1-G2 | I | T1bN0M0 |
| 18 | M | 58 | Kidney | clear cell carcinoma | G2 | I | T1bN0M0 |
| 19 | M | 75 | Kidney | clear cell carcinoma | G1-G2 | I | T1bN0M0 |
| 20 | M | 39 | Kidney | clear cell carcinoma | G2 | I | T1bN0M0 |
| 21 | M | 75 | Kidney | clear cell carcinoma | G2 | I | T1bN0M0 |
| 22 | M | 55 | Kidney | clear cell carcinoma | G1-G2 | I | T1bN0M0 |
| 23 | F | 72 | Kidney | clear cell carcinoma | G2 | I | T1bN0M0 |
| 24 | F | 51 | Kidney | clear cell carcinoma | G2 | I | T1bN0M0 |
| 25 | M | 73 | Kidney | clear cell carcinoma | G2 | I | T1bN0M0 |
| 26 | M | 60 | Kidney | clear cell carcinoma | G1-G2 | II | T2aN0M0 |
| 27 | M | 53 | Kidney | clear cell carcinoma | G2 | II | T2aN0M0 |
| 28 | F | 62 | Kidney | clear cell carcinoma | G3 | II | T2aN0M0 |
| 29 | F | 50 | Kidney | clear cell carcinoma | G2 | I | T1bN0M0 |
| 30 | M | 78 | Kidney | clear cell carcinoma | G2 | I | T1bN0M0 |
| 31 | F | 65 | Kidney | clear cell carcinoma | G3 | I | T1bN0M0 |
| 32 | F | 75 | Kidney | clear cell carcinoma | G3 | I | T1bN0M0 |
| 33 | F | 36 | Kidney | clear cell carcinoma | G1-G3 | II | T2aN0M0 |
| 34 | M | 55 | Kidney | clear cell carcinoma | G2 | II | T2aN0M0 |
| 35 | F | 48 | Kidney | clear cell carcinoma | G3 | II | T2aN0M0 |
| 36 | F | 48 | Kidney | clear cell carcinoma | G1-G2 | II | T2aN0M0 |
| 37 | F | 54 | Kidney | clear cell carcinoma | G2 | II | T2aN0M0 |
| 38 | M | 57 | Kidney | clear cell carcinoma | G1-G4 | II | T2aN0M0 |
| 39 | F | 37 | Kidney | clear cell carcinoma | G1-G2 | II | T2aN0M0 |
| 40 | F | 62 | Kidney | clear cell carcinoma | G2 | II | T2aN0M0 |
| 41 | F | 70 | Kidney | clear cell carcinoma | G2 | II | T2aN0M0 |
| 42 | M | 52 | Kidney | clear cell carcinoma | G2-G3 | II | T2aN0M0 |
| 43 | M | 63 | Kidney | clear cell carcinoma | G2 | II | T2aN0M0 |
| 44 | F | 58 | Kidney | clear cell carcinoma | G2-G3 | II | T2aN0M0 |
| 45 | F | 47 | Kidney | clear cell carcinoma | G3 | II | T2aN0M0 |
| 46 | M | 56 | Kidney | clear cell carcinoma | G2 | II | T2aN0M0 |
| 47 | M | 58 | Kidney | clear cell carcinoma | G2 | II | T2aN0M0 |
| 48 | F | 42 | Kidney | clear cell carcinoma | G2 | II | T2aN0M0 |
| 49 | F | 38 | Kidney | clear cell carcinoma | G3 | II | T2bN0M0 |
| 50 | M | 56 | Kidney | clear cell carcinoma | G2-G3 | III | T3N0M0 |
| 51 | M | 82 | Kidney | clear cell carcinoma | G2 | III | T3N0M0 |
| 52 | M | 77 | Kidney | clear cell carcinoma | G1-G2 | III | T3N0M0 |
| 53 | F | 54 | Kidney | clear cell carcinoma | G3 | II | T2bN0M0 |
| 54 | M | 61 | Kidney | clear cell carcinoma | G2 | II | T2bN0M0 |
| 55 | M | 60 | Kidney | clear cell carcinoma | G2 | II | T2bN0M0 |
| 56 | M | 68 | Kidney | clear cell carcinoma | G2 | III | T3N0M0 |
| 57 | M | 70 | Kidney | clear cell carcinoma | G2 | III | T3N0M0 |
| 58 | M | 65 | Kidney | clear cell carcinoma | G2 | III | T3N0M0 |
| 59 | M | 40 | Kidney | clear cell carcinoma | G1-G2 | III | T3N0M0 |
| 60 | F | 63 | Kidney | clear cell carcinoma | G2-G3 | III | T3N0M0 |
| 61 | M | 56 | Kidney | clear cell carcinoma | G1-G2 | III | T3N0M0 |
| 62 | M | 65 | Kidney | clear cell carcinoma | G2 | III | T3N0M0 |
| 63 | M | 58 | Kidney | clear cell carcinoma | G2 | III | T3N0M0 |
| 64 | M | 56 | Kidney | clear cell carcinoma | G2 | III | T3N0M0 |
| 65 | M | 57 | Kidney | clear cell carcinoma | G2-G3 | III | T3N0M0 |
| 66 | M | 51 | Kidney | clear cell carcinoma | G2 | IV | T4N0M0 |
| 67 | F | 49 | Kidney | clear cell carcinoma | G2 | IV | T4N0M0 |
| 68 | M | 74 | Kidney | clear cell carcinoma | G2 | IV | T4N0M0 |
| 69 | M | 70 | Kidney | clear cell carcinoma | G2-G3 | III | T3N0M0 |
| 70 | M | 58 | Kidney | clear cell carcinoma | G2-G3 | III | T2aN1M0 |
| 71 | F | 58 | Kidney | clear cell carcinoma | G3-G4 | III | T2bN1M0 |
| 72 | F | 58 | Kidney | clear cell carcinoma | G3 | III | T3N1M0 |
| 73 | M | 60 | Kidney | clear cell carcinoma | G2-G3 | IV | T4N0M0 |
| 74 | M | 73 | Kidney | clear cell carcinoma | G2-G3 | IV | T1b? M1 |
| 75 | M | 56 | Kidney | clear cell carcinoma | G2 | IV | T3N0M1 |
